# Expression of PTP4A1 in circulating tumor cells and its efficacy evaluation in patients with early- and intermediate-stage esophageal cancer

**DOI:** 10.1097/MD.0000000000036603

**Published:** 2023-12-22

**Authors:** Jie Wu, Wen-Xiang Wang, Yong Zhou, De-Song Yang, Zhi-Ning Wu, Xu Li, Jin-Ming Tang

**Affiliations:** a Thoracic Surgery Department II, Hunan Provincial Tumor Hospital, Changsha, Hunan, China; b Thoracic Surgery Department I, Hunan Provincial Tumor Hospital, Changsha, Hunan, China.

**Keywords:** circulating tumor cells, esophageal cancer, lymph node metastasis, PTP4A1, TNM staging

## Abstract

The objective of this study is to investigate the expression of protein tyrosine phosphatase type I (PTP4A1) in circulating tumor cells (CTCs) in patients with early- and intermediate-stage esophageal cancer and its clinical value in evaluating patient prognosis. Tissue and peripheral blood samples were collected from patients with esophageal cancer, as well as their clinical data. Follow-up was performed on all patients. PTP4A1 expression in the CTCs of patients were analyzed by regression analysis, and its correlation with the clinical characteristics of esophageal cancer was discussed. The numbers of mixed tumor cells and T-CTCs were significantly correlated with lymph node metastasis. Advanced tumor-node metastasis (TNM) stage (odds ratio = 12.063) and lymph node metastasis (odds ratio = 13.541) were influencing factors of PTP4A1^+^MCTC expression disorders in patients with esophageal cancer. The receiver operating characteristic curve showed that TNM stage and lymph node metastasis had a high predictive efficiency for PTP4A1^+^MCTCs, with an area under the ROC curve of 0.725. PTP4A1^+^mixed tumor cells had strong predictive value for the efficacy of neoadjuvant therapy, with a sensitivity of 94.7% and a specificity of 63.6%. Advanced TNM stage and lymph node metastasis are influencing factors for increased CTCs and poor expression of PTP4A1 in patients with esophageal cancer.

## 1. Introduction

Esophageal cancer is a malignancy occurring in the esophagus with relatively high incidence and fatality rates. According to global cancer statistics, esophageal cancer is the sixth most common and seventh deadliest gastrointestinal malignant tumor.^[1,2]^ Despite advances in treatment methods in recent years, esophageal cancer has shown a rising incidence due to its highly aggressive and metastatic nature, resulting in poor patient outcomes with a 5-year survival rate of <20%.^[3]^ Therefore, it is particularly urgent to understand the pathogenesis of esophageal cancer and find new early diagnostic markers. Circulating tumor cells (CTCs) are a general term for all kinds of tumor cells in peripheral blood. Relevant studies have reported that CTCs are an independent prognostic factor for a variety of solid tumors and can significantly increase the death risk of patients with malignant tumors.^[4]^ Protein tyrosine phosphatase type IVA (member 1, PTP4A1) is a nuclear protein that is highly expressed in a variety of cancers, such as colorectal, prostate, breast, stomach and hepatocellular carcinomas.^[5]^ It has been found that PTP4A1 plays an important role in the occurrence and development of various tumors by mediating cell growth and tumor development through various signaling pathways and, to some extent, promoting tumor formation, proliferation, and metastasis. International studies on the expression of PTP4A1 based on CTCs are mostly found in lung cancer, but no relevant reports have been reported in esophageal cancer.^[6,7]^ Therefore, this study explored the factors influencing treatment efficacy in patients with esophageal cancer and constructed a prediction model to investigate the value of PTP4A1 expression combined with CTCs in evaluating the occurrence, development, and prognosis of patients with esophageal cancer, thus providing a reliable reference for clinical decision making.

## 2. Materials and methods

### 2.1. Subjects

A total of 99 patients with early- or intermediate-stage esophageal cancer admitted to the Hunan Provincial Tumor Hospital between January 2021 and April 2023 were selected as research subjects. The inclusion criteria were as follows: patients (age: ≥18 years and ≤75 years) diagnosed with stage I–III esophageal cancer by pathological diagnosis^[8]^ who underwent surgery in our hospital and pre- and postoperative peripheral blood sampling (5 mL × 2 tubes of cubital venous blood), with no previous antineoplastic therapy. The following were the exclusion criteria: age <18 or >75; history of other malignant tumors; patients who received new targeted drugs not marketed in China through various channels; severe liver and renal function damage, or severe cardiac insufficiency; active infection, uncorrectable coagulopathy, obvious abnormal blood picture, or obvious bleeding tendency; those who failed to follow up and reexamine on time for various reasons; history of craniocerebral injury or trauma; loss to follow-ups.

### 2.2. Methodology

#### 2.2.1. Collection of samples.

Using an 8-gauge needle, a peripheral blood sample (2 × 5 mL) was collected from each patient into an ethylene diamine tetraacetic acid anticoagulant tube, which was then transferred to the sample storage tube for pretreatment.

#### 2.2.2. Detection of CTCs and PTP4A1 gene expression.

The CanPatrol CTC detection system was used to detect the number and type of CTCs and the expression of PTP4A1 in the peripheral blood of patients with esophageal cancer. A total of 99 peripheral blood samples were analyzed. The specific steps were as follows: ① Peripheral blood samples were collected using 8-gauge needles and ethylene diamine tetraacetic acid anticoagulant tubes. ② The samples were then transferred to the sample storage tube for pretreatment, followed by filtering into the filter. ③ Markers of PTP4A1 and CTCs were used to capture the probe and hybridize the target mRNAs. ④ The amplification probe was hybridized with the capture probe to prepare for the amplification of hybridization signals. ⑤ A probe labeled with fluorophores (purple, red, and green fluorescent labels) was hybridized with an amplified probe to generate a fluorescent signal, and the nucleus was labeled with diamidino-2-phenylindole staining (blue fluorescent label). ⑥ The automatic identification system was used to read the fluorescence signal and automatically judge the detection result. ⑦ CTCs and CTC-PTP4A1 were detected within 1 week before surgery, 7 days after surgery, and 1 month after surgery to monitor tumor recurrence and metastasis.

### 2.3. Follow-up

Patients were followed-up for 3 months by reviewing hospital records and contacting patients and their families. Overall survival was defined as the time from the date of surgery to the end of follow-up or the date of death from esophageal cancer. Records of medical record information: According to the tumor-node-metastasis (TNM) grading criteria of the World Health Organization, the clinicopathological information of all the enrolled patients was recorded and analyzed, including patient age, sex, tumor size, tumor grade, lymph node metastasis, degree of differentiation, pathological response, tumor recurrence, and death. Observation termination events included death, withdrawal from treatment, request for withdrawal from the study, and observed time endpoint.

### 2.4. Statistical analysis

The SPSS27.0 statistical software (IBM, Armonk, NY) was used to analyze the collected data. Logistic regression analysis was used to analyze the factors influencing treatment efficacy in patients with esophageal cancer. The chi-square test, Fisher exact test, or Mann–Whitney *U* test was used to compare the distribution differences between groups. All tests were 2-tailed with the test level set at α = 0.05. The predictive value of the related factors was evaluated using the receiver operating characteristic (ROC) curve.

## 3. Results

### 3.1. CTC typing and PTP4A1 expression results

**Figure 1.**
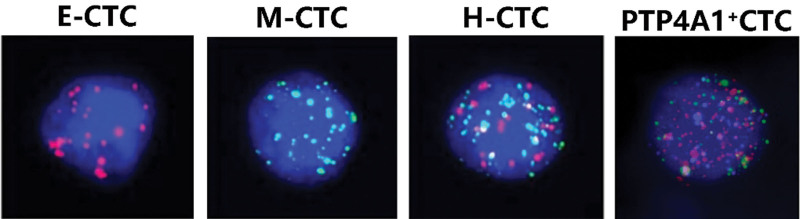
CTC typing and staining of PTP4A1-positive cells. E-CTC = epithelial tumor cells, H-CTC = mixed tumor cells, M-CTC = mesenchymal tumor cells.

The CanPatrol technique can successfully isolate and classify CTCs simultaneously. CTC typing and pictures of PTP4A1 positive CTCs are shown in Figure 1, where E-CTCs, M-CTCs, mixed tumor cells (H-CTCs), and PTP4A1^+^CTCs are indicated by red, green, red and green, and purple fluorescent signal spots, respectively.

### 3.2. Analysis of clinical characteristics of different CTC subtypes

The total number of CTCs (T-CTCs) was significantly correlated with clinical staging (TNM) and lymph node metastasis. The number of samples with T-CTCs ≥ 5 in the TNM stage was significantly higher than that with T-CTCs < 5 (*P* = .006), and the number of samples with T-CTCs ≥ 5 in the group with lymph node metastasis was significantly higher (*P* = .040). The number of M-CTCs was related to lymph node metastasis, with a significantly higher M-CTC count in the lymph node metastasis group compared with the non-lymph node metastasis group (*P* = .047) (Table 1).

**Table 1:** Analysis of different CTC subtypes and clinical characteristics of patients.

Characteristics	T-CTCs		*P*	E-CTCs		*P*	M-CTCs		*P*	H-CTCs		*P*
	<5	≥5		<1	≥1		<1	≥1		<1	≥1	
Age (yr)												
<60	11	36	.451	7	40	.568	21	26	.735	15	32	.740
≥60	9	43		10	42		25	27		15	37	
Sex												
Male	17	72	.418	15	74	.680	42	47	.748	27	62	1.000
Female	3	7		2	8		4	6		7	3	
Tumor size (cm)												
<3	10	44	.648	9	45	.884	26	28	.713	17	37	.780
≥3	10	35		8	37		20	25		13	32	
TNM (stages)												
I-II	16	36	**.006**	8	44	.620	28	24	.121	18	34	.326
III	4	43		9	38		18	29		12	35	
Lymph node metastasis												
Yes	5	40	**.040**	7	38	.697	16	29	**.047**	12	33	.472
No	15	39		10	44		30	24		18	36	
Differentiation												
Low	5	23	.894	6	22	.662	10	18	.372	8	20	.829
Middle	10	35		8	37		22	23		15	30	
High	5	21		3	23		14	12		7	19	

CTCs = circulating tumor cells.

Based on the above results, the correlation between TNM staging, lymph node metastasis, and CTC subtype was analyzed. The results showed that the number of H-CTCs and T-CTCs in TNM stage III was significantly higher than that in TNM stages I-II (*P* = .091, *P* = .045). The H-CTC and T-CTC counts were also significantly correlated with lymph node metastasis (*P* = .045 and *P* = .013, respectively).

### 3.3. Analysis of the correlation between PTP4A1 expression in CTCs and patient clinical characteristics

The results showed that PTP4A1^+^MCTCs were significantly correlated with clinical TNM staging and lymph node metastasis (*P* = .036 and *P* = .013, respectively). Neither PTP4A1^+^ECTCs nor PTP4A1^+^HCTCs showed significant correlation with the included clinical indicators, as shown in Table 2.

**Table 2:** PTP4A1 expression in different CTC subtypes and analysis of patient clinical characteristics.

Characteristics	PTP4A1^+^ECTCs		*P* value	PTP4A1^+^MCTCs		*P* value	PTP4A1^+^HCTCs		*P* value
	<1	≥1		<1	≥1		<1	≥1	
Age (yr)									
<60	14	33	.915	24	23	.932	18	29	.560
≥60	16	36		27	25		17	35	
Sex									
Male	26	63	.485	46	43	1.000	32	57	1.000
Female	4	6		5	5		3	7	
Tumor size (cm)									
<3	17	37	.780	29	25	.633	20	34	.701
≥3	13	32		22	23		15	30	
TNM (stages)									
I-II	18	34	.326	32	20	**.036**	22	30	.128
III	12	35		19	28		13	34	
Lymph node metastasis									
Yes	12	33	.472	17	28	**.013**	15	30	.701
No	18	36		34	20		20	34	
Differentiation									
Low	8	20	.530	12	16	.551	9	19	.883
Middle	16	29		25	20		17	28	
High	6	20		14	12		9	17	

CTCs = circulating tumor cells, PTP4A1 = protein tyrosine phosphatase type I.

Based on the above single factor results, multivariate regression analysis of the expression level of PTP4A1^+^MCTCs revealed that advanced TNM stage (odds ratio, OR = 12.063) and lymph node metastasis (OR = 13.541) were influencing factors of PTP4A1^+^MCTCs expression disorders in esophageal cancer patients (*P* < .05), as shown in Table 3.

**Table 3:** Multivariate regression analysis.

	Factor	*β*	S.E.	Wald *χ*²	*P* value	OR	95% CI
PTP4A1^+^MCTCs	TNM staging	2.49	0.775	10.332	<.001	12.063	2.643–55.066
	Lymph node metastasis	2.303	0.776	11.277	<.001	13.541	2.959–61.965

CI = confidence interval, OR = odds ratio.

### 3.4. Construction and validation of the prediction model

**Figure 2.**
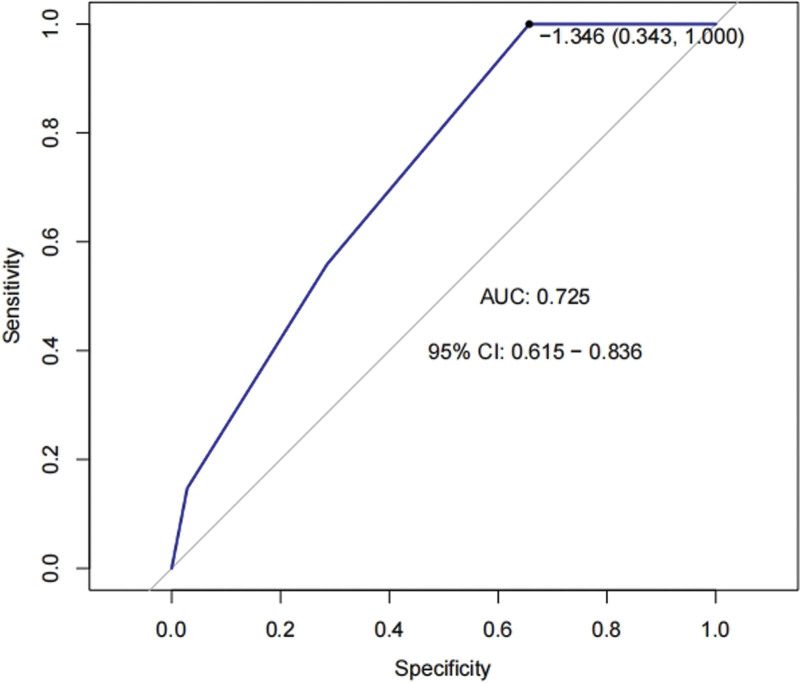
ROC curve of the prediction mode. ROC = receiver operating characteristic.

From the above, we can see that patients with an advanced TNM stage and lymph node metastasis presented low expression levels of PTP4A1^+^MCTCs. Therefore, we constructed a prediction model to explore the predictive efficacy of TNM staging and lymph node metastasis for PTP4A1^+^MCTCs and plotted ROC curves. The area under the ROC curve (AUC) was found to be 0.725, indicating good predictive performance of the model, as shown in Figure 2.

### 3.5. Correlation of CTC subtypes and PTP4A1 expression with the efficacy of neoadjuvant therapy in esophageal cancer

Seventy-four of the 99 patients received neoadjuvant therapy and some experienced adverse reactions. Among them, M-CTCs, PTP4A1^+^ECTCs, and PTP4A1^+^MCTCs were found to be correlated with adjuvant therapy and affected patients’ prognosis (*P* < .05), as shown in Table 4.

**Table 4:** Correlation of CTC subtypes and PTP4A1 expression with the efficacy of neoadjuvant therapy in esophageal cancer.

	The efficacy of neoadjuvant therapy		*P*
	Good	Poor efficacy	
T-CTCs			
<5	5	10	.513
≥5	14	45	
E-CTCs			
<1	3	11	1.000
≥1	16	44	
M-CTCs			
<1	14	18	.002
≥1	5	37	
H-CTCs			
<1	5	17	.706
≥1	14	38	
PTP4A1^+^ECTCs			
<1	10	15	.044
≥1	9	40	
PTP4A1^+^MCTCs			
<1	15	20	.001
≥1	4	35	
PTP4A1^+^HCTCs			
<1	8	11	.374
≥1	17	38	

CTCs = circulating tumor cells, PTP4A1 = protein tyrosine phosphatase type I.

**Figure 3.**
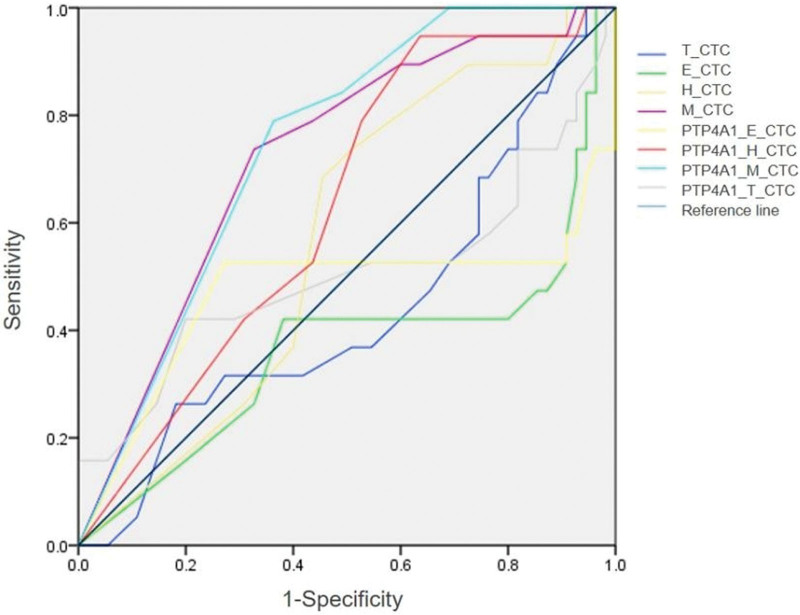
The expression of different subtypes of CTCs and PTP4A1 predicts the efficacy of neoadjuvant therapy in esophageal cancer. CTCs = circulating tumor cells, PTP4A1 = protein tyrosine phosphatase type I.

In addition, the cell counts of different CTC subtypes and PTP4A1 expression were used to establish a predictive model for response to neoadjuvant therapy. The ROC curve analysis showed that M-CTCs had the strongest predictive power for the efficacy of neoadjuvant therapy among all CTC subtypes, with an AUC of 0.720, a sensitivity of 73.70%, and a specificity of 32.70%. The AUC, sensitivity, and specificity of H-CTCs were 0.570, 68.40%, and 45.50%, respectively. To improve prediction efficiency, we reestablished a prediction model based on the expression of PTP4A1. The results showed that the sensitivity of PTP4A1^+^MCTCs increased to 78.90%, but the specificity decreased to 32.70%. The sensitivity and specificity of PTP4A1^+^HCTCs increased to 94.7% and 63.6%, respectively (Fig. 3). In conclusion, the combination of H-CTCs and PTP4A1 expression can help predict the efficacy of neoadjuvant therapy in patients with esophageal cancer more sensitively and specifically.

## 4. Discussion

Esophageal cancer, a malignant tumor originating from the esophageal mucosal epithelial tissue, is a malignant disease with multi-factor participation, multi-stage development, and multi-gene changes, with high morbidity and mortality.^[9]^ In recent years, advances in surgical treatment, radiotherapy, and chemotherapy for esophageal cancer have prolonged the survival of patients and enhanced their quality of life. However, some patients still experience adverse reactions after treatment, with a high postoperative recurrence rate and poor prognosis.^[10]^ Relevant studies have found that CTCs are an independent risk factor for adverse prognosis in patients with esophageal cancer and are the most direct factor for tumor metastasis and recurrence.^[11,12]^ PTP4A1 is a relatively new gene with varying degrees of expression in different tumor tissues and cell lines and is related to tumorigenesis and metastasis.^[13]^ Studies have shown that the inhibition of endogenous PTP4A1 in tumor cells in mouse models can cause loss of cell motility and metastasis.^[14]^ Therefore, it is speculated that PTP4A1 has the potential to be a biomarker for tumor recurrence and metastasis and a target for tumor therapy. However, there are few studies investigating its expression and mechanism in esophageal cancer. Accordingly, this study explored the factors related to recurrence and metastasis in patients with esophageal cancer and established a predictive model using different CTC subtypes and PTP4A1 expression to evaluate the predictive efficacy of PTP4A1 for the treatment of esophageal cancer.

Common clinical diagnostic modalities for tumors, which mainly include imaging, serology, and pathology, cannot be comprehensively monitored in real-time to obtain tumor information, resulting in poor prognosis in some patients. The CanPatrol CTC detection technique, on the other hand, effectively overcomes the limitation of first-generation technology that can only isolate CTCs with a specific epithelial phenotype. It uses nano-filtration to separate CTCs and simultaneously realize the isolation, identification, and classification of single cell CTCs and Cell mass (also known as circulating tumor microembolus), enabling dynamic real-time monitoring of tumors. CanPatrol technology is commonly used in the clinical practice of liver cancer, and the treatment methods include surgery, chemotherapy, transcatheter arterial chemoembolization, and percutaneous radiofrequency ablation. In this study, CanPatrol CTC detection technology was used to isolate and classify CTCs simultaneously, contributing to a high positive rate. The development of this technique in other fields is further advanced.

The results of this study showed that both CTC subtypes and PTP4A1 expression levels were associated with the TNM stage and lymph node metastasis of tumors. It is suggested that advanced stages of TNM and lymph node metastasis will increase the peripheral blood CTC count and hinder the expression of PTP4A1, thereby affecting the prognosis of patients with esophageal cancer. TNM is currently a popular tumor-staging system. Patients with an advanced TNM stage are at increased risk of recurrence after treatment, resulting in poor treatment efficacy. Related studies have found that CTC-positive patients have a later TNM stage and worse prognosis, consistent with the results of this study.^[11,15,16]^ Similarly, lymph node metastasis is a risk factor for local recurrence, distant metastasis, and reduced survival. Therefore, preoperative evaluation of lymph node metastasis is of great significance for the choice of surgical modality, decrease of the postoperative recurrence rate, and reduction of surgical complications. The distribution of CTC subtypes is shown to be significantly related to the status of lymph node metastasis.^[17,18]^ PTP4A1 was first identified as an early stimulus response factor involved in the regulation of cyclin phosphorylation and plays an important role in cell growth.^[7]^ Previous studies have shown that microRNAs regulate cancer metastasis. Li Chao et al found that microRNAs were related to advanced TNM differentiation, and PTP4A1-mediated phosphorylation was identified as a direct downstream target of microRNAs.^[6,19]^ Tao et al reported that PTP4A1 was related to the status of lymph node metastasis, and the increase in PTP4A1 expression would cause an increase in the tumor recurrence rate, affecting patient outcomes and reducing patient survival.^[7]^

In addition, this study found that PTP4A1 in combination with CTCs was highly predictive of the efficacy of adjuvant therapy in patients with esophageal cancer. PTP4A1 is a member of the regenerating growth factor family of liver phosphatases, a subclass of the protein tyrosine phospholipase family, which affects the occurrence and progression of tumors.^[20]^ Abnormally expressed PTP4A1 is closely related to the malignant biological behaviors of tumor cells, such as anoikis resistance, invasion and metastasis, epithelial-mesenchymal transition, and angiogenesis.^[21]^ Knockout of the PTP4A1 gene in small cell lung cancer has been indicated to lower the expression of c-Src to reduce cell proliferation, adhesion, and invasion, further demonstrating that highly expressed PTP4A1 protein in esophageal cancer will promote the occurrence and development of tumors, and affecting the postoperative recurrence and metastasis of patients.^[22]^

This study has certain limitations. First, the sample size included is insufficient due to the limited number of esophageal cancer patients admitted to our hospital, which affects the results to some extent. Second, this study considers few clinical features and only explores relevant information such as tumor size and degree of differentiation. Based on the results of this study, the sample size will be further expanded in the future and other related indicators of esophageal cancer patients will be included to explore the influencing factors of poor prognosis, so as to provide more reliable evidence for clinical research. Third, multiple prediction models will be established based on the expression of PTP4A1 in CTCs to predict lymph node metastasis and efficacy of neoadjuvant therapy in patients with esophageal cancer.

## 5. Conclusion

In conclusion, advanced TNM stage and lymph node metastasis are factors influencing the increase in CTCs and the blockage of PTP4A1 expression in patients with esophageal cancer. CTCs combined with PTP4A1 expression can help better predict treatment efficacy in patients with esophageal cancer, which has important clinical value in blocking the disease progression and achieving individualized treatment of esophageal cancer metastasis.

## Author contributions

**Conceptualization:** Jie Wu, Jin-Ming Tang.

**Data curation:** Jie Wu, Wen-Xiang Wang, Jin-Ming Tang.

**Formal analysis:** Wen-Xiang Wang, Zhi-Ning Wu.

**Funding acquisition:** Jie Wu, De-Song Yang, Zhi-Ning Wu, Xu Li, Jin-Ming Tang.

**Investigation:** De-Song Yang, Jin-Ming Tang.

**Methodology:** Jie Wu, Wen-Xiang Wang.

**Project administration:** Yong Zhou.

**Resources:** Jie Wu, Wen-Xiang Wang, Yong Zhou, De-Song Yang, Zhi-Ning Wu.

**Software:** Jie Wu, Wen-Xiang Wang, Yong Zhou.

**Supervision:** Xu Li, Jin-Ming Tang.

**Validation:** Jie Wu, Wen-Xiang Wang, De-Song Yang, Jin-Ming Tang.

**Visualization:** Jie Wu.

**Writing – original draft:** Jie Wu, Jin-Ming Tang.

**Writing – review & editing:** Jie Wu, Jin-Ming Tang.
